# Piezochromism and hydrochromism through electron transfer: new stories for viologen materials†
†Electronic supplementary information (ESI) available: Synthetic procedures, bond data. CCDC 1428498. For ESI and crystallographic data in CIF or other electronic format see DOI: 10.1039/c6sc04579k



**DOI:** 10.1039/c6sc04579k

**Published:** 2016-12-22

**Authors:** Qi Sui, Xiang-Ting Ren, Yu-Xiang Dai, Kai Wang, Wen-Tao Li, Teng Gong, Jia-Jia Fang, Bo Zou, En-Qing Gao, Lin Wang

**Affiliations:** a Shanghai Key Laboratory of Green Chemistry and Chemical Processes , College of Chemistry and Molecular Engineering , East China Normal University , 3663 North Zhongshan Road , Shanghai 200062 , P. R. China . Email: eqgao@chem.ecnu.edu.cn; b Center for High Pressure Science and Technology Advanced Research , 1690 Cailun Road , Shanghai 201203 , P. R. China . Email: wanglin@hpstar.ac.cn; c State Key Laboratory of Superhard Materials , Jilin University , 2699 Qianjin Street , Changchun , Jilin 130012 , P. R. China

## Abstract

While viologen derivatives have long been known for electrochromism and photochromism, here we demonstrated that a viologen-carboxylate zwitterionic molecule in the crystalline state exhibits piezochromic and hydrochromic behaviors. The yellow crystal undergoes a reversible color change to red under high pressure, to green after decompression, and finally back to yellow upon standing at ambient pressure. Ultraviolet-visible spectroscopy, X-ray photoelectron spectroscopy, electron paramagnetic resonance X-ray diffraction and DFT calculations suggested that the piezochromism is due to the formation of radicals *via* pressure-induced electron transfer from carboxylate to pyridinium, without a crystallographic phase transition. It was proposed that electron transfer is induced by pressure-forced reduction of intermolecular donor–acceptor contacts. The electron transfer can also be induced by dehydration, which gives a stable green anhydrous radical phase. The color change is reversible upon reabsorption of water, which triggers reverse electron transfer. The compound not only demonstrates new chromic phenomena for viologen compounds, but also represents the first example of organic mechanochromism and hydrochromism associated with radical formation *via* electron transfer.

## Abstract 2

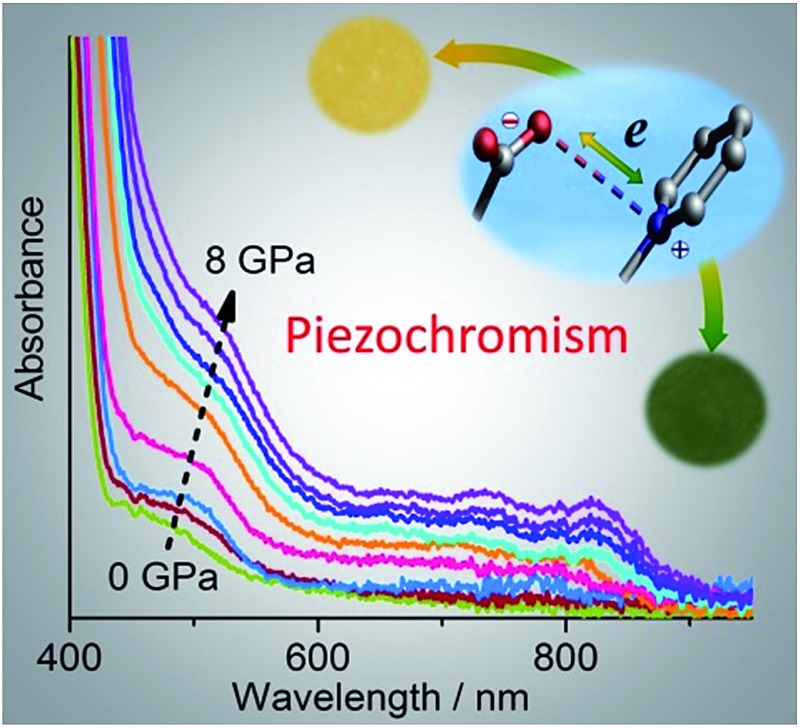
A pyridinium-carboxylate compound undergoes reversible color change under pressure owing to the formation of radicals *via* electron transfer; dehydration and hydration can also trigger electron transfer.

## Introduction

Chromic materials that change color in response to external physical or chemical stimuli have been enduring topics of research for a variety of present and potential applications in our daily life and in various high-tech areas.^1^ In general, the color change is related to a stimulus-induced change in electronic structure, for example, decreased band-gaps for semiconductors,^2^ or modulated ligand-fields for d-block compounds.^3^ For organic molecules, the change is often caused by structural isomerization (such as ring-opening/closing,^4^ *cis*–*trans* isomerization,^5^ and tautomerism^6^), free-radical formation^7^ or just conformational changes.^8^ Various chromic phenomena, named after the stimuli, such as photochromism, electrochromism, mechanochromism and solvatochromism, have been actively studied. Piezochromism, a subclass of mechanochromism,^9^ refers to the reversible color change of a solid in response to external pressure. Recently there have been intense studies on piezochromic luminescence, where pressure induced a change in the photoluminescence color of the material.^10^ In this paper, we are focused on innocent piezochromism, where pressure can induce a change in the natural color of the material. The phenomenon has been observed for various materials, including metal oxides/complexes (such as molybdates,^11^ Cu(ii) complexes,^2a,12^ Ni(ii) glyoximates^13^ and Fe(ii) spin-crossover compounds^14^), polymers (such as polysilanes^15^ and polythiophenes^16^), organic molecules in polymer matrices,^17^ and crystalline organic molecular solids.^18^

Since covalent bonds are rather resistant to the effects of pressure, organic molecular solids respond to high pressure mainly by reducing intermolecular space and/or changing molecular conformations (if flexible).^19^ In the presence of appropriate chromophores, the pressure-forced structural changes may lead to a color change through modulation of electronic energy gaps. Some flexible organic molecules with π-conjugation or donor–acceptor chromophores have been reported to exhibit piezochromism in crystalline states, where pressure enhances intermolecular π overlap or intramolecular coplanarity and thus leads to bathochromic shifts of π–π* or charge-transfer (CT) absorptions.^8^ In extreme cases, pressure-forced structural changes can result in irreversible inter-/intramolecular chemical transformations such as polymerization and isomerization, concomitant with irreversible (undesirable) color change.^18^ Nevertheless, in a few favorable cases of spiropyrans and analogous compounds, reversible piezochromism has been achieved where pressure induces reversible ring-opening isomerization through C–O bond heterolysis.^18b,20^ Piezochromism related to reversible bond homolysis (C–C or S–S), which leads to colored radicals, has also been reported for a few dimeric compounds (bis-dithiazolyl, bis-thioindoxyl and bis-chromenyl, bis-imidazolyl derivatives).^21^ Such dimer–radical interconversion is also responsible for mechanochromism by grinding (*i.e.*, tribochromism^9^) in some dimeric compounds.^7f–j^

**Scheme 1:**
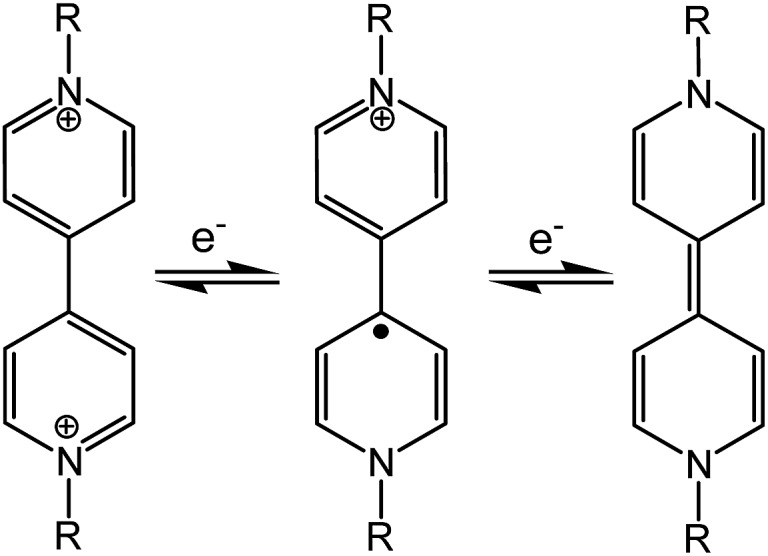
Redox processes of viologens.

Viologen compounds (1,1′-disubstituted 4,4′-bipyridiniums, also often called paraquat derivatives) have long been known for two well-established properties associated with their special electron-deficient attribute:^22^ the propensity to form charge-transfer complexes with electron-rich species, and the redox capability (Scheme 1) to undergo reversible electron transfer under chemical, electrical or optical stimuli. Thanks to these properties, the viologen unit has been widely used in the field of supramolecular chemistry as the key building block for the assembly of mechanically interlocked molecules^23^ and molecular machines.^24^ The reversible redox activity involving brilliantly colored viologen radicals (V˙^+^) has also allowed for widespread applications of viologens in the field of chromic materials. In fact, viologens are the most extensively studied organic electrochromes^1a,22^ and they can also be photochromic in the presence of appropriate electron donors.^25^ In contrast to the well-known electro- and photochromism, we are unaware of any previous reports of mechanochromism related to radical formation through electron transfer, in either viologens or any other class of compounds. In this paper, we demonstrate reversible piezochromism observed with a zwitterionic viologen compound bearing carboxylate groups. The piezochromism involves radical formation/quenching *via* reversible electron transfer between carboxylate and viologen, which could be related to pressure-controlled intermolecular donor–acceptor contacts. Notably, the mechanism for radical formation in the viologen compound (electron transfer) is clearly distinguished from that in previous mechanochromic compounds involving radical formation (bond homolysis).^7f–j,21^

In addition, we show that the compound behaves as a “chromic sponge”: the release and re-absorption of lattice water induce reversible electron transfer and consequent color change. Reversible solid-state hydrochromism related to dehydration–hydration has long been known in transition metal compounds,^26^ among the best known examples being copper sulfate pentahydrate and cobalt nitrate hexahydrate. The phenomenon has also been observed in organic species.^27^ Very recently, some oxazolidines and oxazines were shown to be hydrochromic owing to water-triggered reversible ring-opening isomerisation, and have been tested for water-jet rewritable printing.^28^ To the best of our knowledge, solid-state organic hydrochromism through the electron transfer mechanism is unprecedented.

## Results and discussion

### Crystal structure

The compound, *N*,*N*′-bis(carboxylatophenyl)-4,4′-bipyridinium hexahydrate (bpybdc·6H_2_O, **1**) was synthesized by a multi-step procedure involving the Zincke reaction of *N*,*N*′-bis(2,4-dinitrophenyl)-4,4′-bipyridinium dichloride and ethyl 4-aminobenzoate (Scheme S1†).

**Fig. 1:**
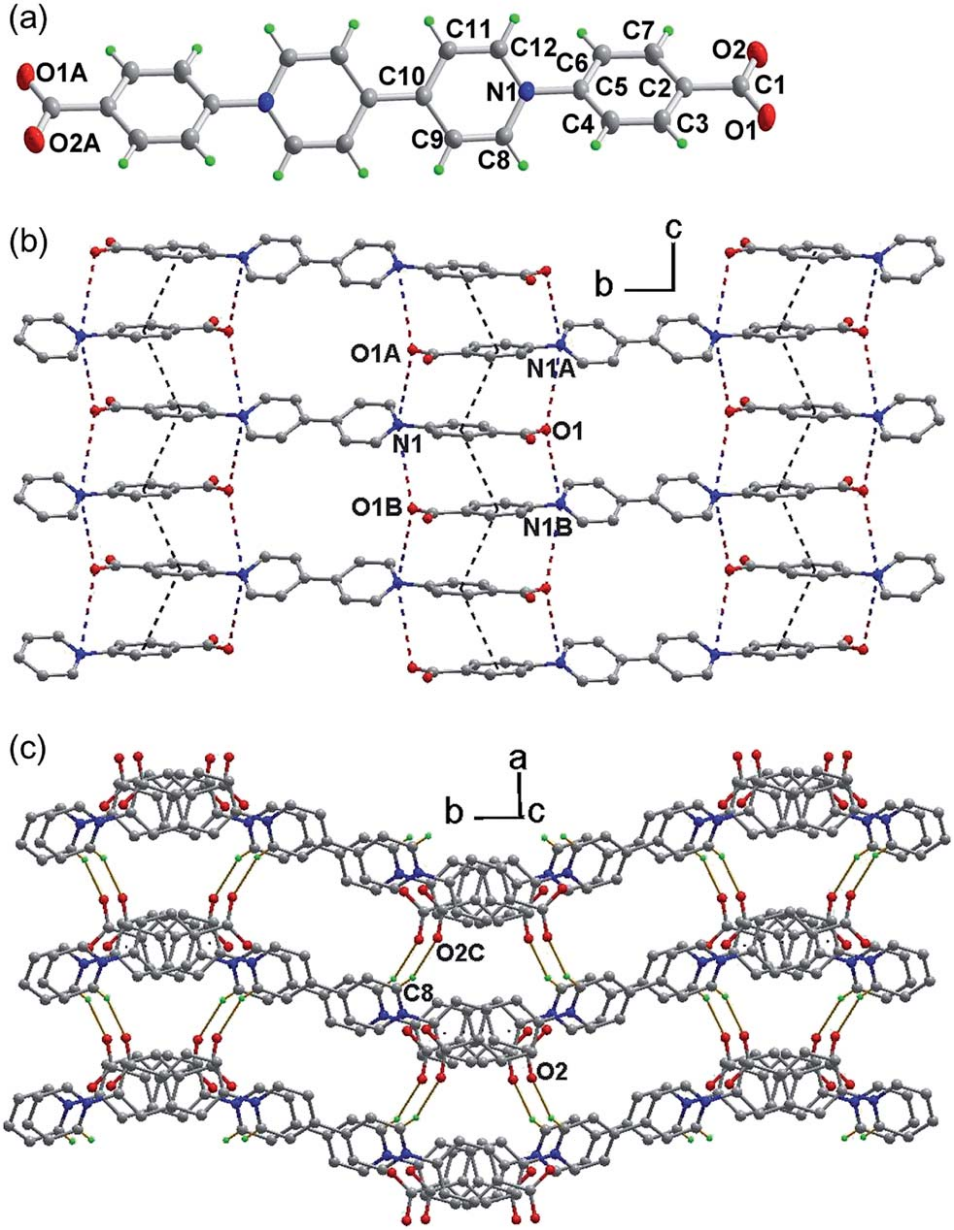
(a) Structure of the zwitterion in **1** (ellipsoids in 50% probability). (b) A 2D hydrogen-bonded layer with intermolecular interactions. (c) Three-dimensional organic network. Symmetry code: *A* = *x*, 0.5 – *y*, 0.5 + *z*; *B* = *x*, 0.5 – *y*, –0.5 + *z*; *C* = 1 + *x*, 0.5 – *y*, 0.5 + *z*.

The structure was determined by single-crystal X-ray crystallography (Fig. 1). The zwitterion bpybdc is centrosymmetric with the central bipyridinium moiety being strictly planar. The terminal benzoate moiety is quasi-planar with a dihedral angle of 2.0(2)° between the benzene ring and the carboxylate group, and the benzene ring is twisted with respect to the bipyridinium plane by a dihedral angle of 33.85(4)°. Neighboring benzoate moieties from different molecules are nearly parallel [dihedral angle: 0.82(2)°] and held together by extended π–π stacking interactions to form a one-dimensional zipper-like array along the *c* direction (Fig. 1b). The average atom-to-plane distance is 3.476(1) Å, and the centroid–centroid distance is 3.818(1) Å between the π–π stacking benzene rings. The π–π stacking interactions, together with the electrostatic force, pull into close proximity the pyridinium and carboxylate groups from different molecules. Each pyridinium N (carboxylate O) atom has two neighboring carboxylate O (pyridinium N) atoms on opposite sides, with the N···O distances being 3.514(2) (N1···O1A) and 3.695(2) Å (N1···O1B). Through the above intermolecular interactions, the zwitterionic molecules are zippered into a waved sheet along the *bc* plane. The sheets are assembled into a three-dimensional organic network through hydrogen bonding interactions between carboxylate O and pyridinium H atoms from neighboring sheets [Fig. 1C. C8–H8···O2C, with <svg xmlns="http://www.w3.org/2000/svg" version="1.0" width="16.000000pt" height="16.000000pt" viewBox="0 0 16.000000 16.000000" preserveAspectRatio="xMidYMid meet"><metadata> Created by potrace 1.16, written by Peter Selinger 2001-2019 </metadata><g transform="translate(1.000000,15.000000) scale(0.005147,-0.005147)" fill="currentColor" stroke="none"><path d="M2560 2120 l0 -40 -160 0 -160 0 0 -40 0 -40 -160 0 -160 0 0 -40 0 -40 -160 0 -160 0 0 -40 0 -40 -160 0 -160 0 0 -40 0 -40 -160 0 -160 0 0 -40 0 -40 -160 0 -160 0 0 -40 0 -40 -160 0 -160 0 0 -40 0 -40 -160 0 -160 0 0 -80 0 -80 160 0 160 0 0 40 0 40 160 0 160 0 0 40 0 40 160 0 160 0 0 40 0 40 160 0 160 0 0 40 0 40 160 0 160 0 0 40 0 40 160 0 160 0 0 40 0 40 160 0 160 0 0 40 0 40 160 0 160 0 0 40 0 40 80 0 80 0 0 80 0 80 -80 0 -80 0 0 -40z M0 960 l0 -80 160 0 160 0 0 -40 0 -40 160 0 160 0 0 -40 0 -40 160 0 160 0 0 -40 0 -40 160 0 160 0 0 -40 0 -40 160 0 160 0 0 -40 0 -40 160 0 160 0 0 -40 0 -40 160 0 160 0 0 -40 0 -40 160 0 160 0 0 -40 0 -40 80 0 80 0 0 80 0 80 -80 0 -80 0 0 40 0 40 -160 0 -160 0 0 40 0 40 -160 0 -160 0 0 40 0 40 -160 0 -160 0 0 40 0 40 -160 0 -160 0 0 40 0 40 -160 0 -160 0 0 40 0 40 -160 0 -160 0 0 40 0 40 -160 0 -160 0 0 40 0 40 -160 0 -160 0 0 -80z"/></g></svg> C–H···O = 158.14(8)°, H···O = 2.286(1) Å and C···O = 3.167(2) Å]. The intermolecular N···O and C–H···O contacts between donor and acceptor groups have been considered to be important parameters for photochromism of viologen-based organic and metal–organic solids.^7d,29^ Lattice water molecules are embedded among the organic network *via* O–H···O and C–H···O interactions (Table S1 and Fig. S1†).

### Piezochromism under uniaxial pressure

**Fig. 2:**
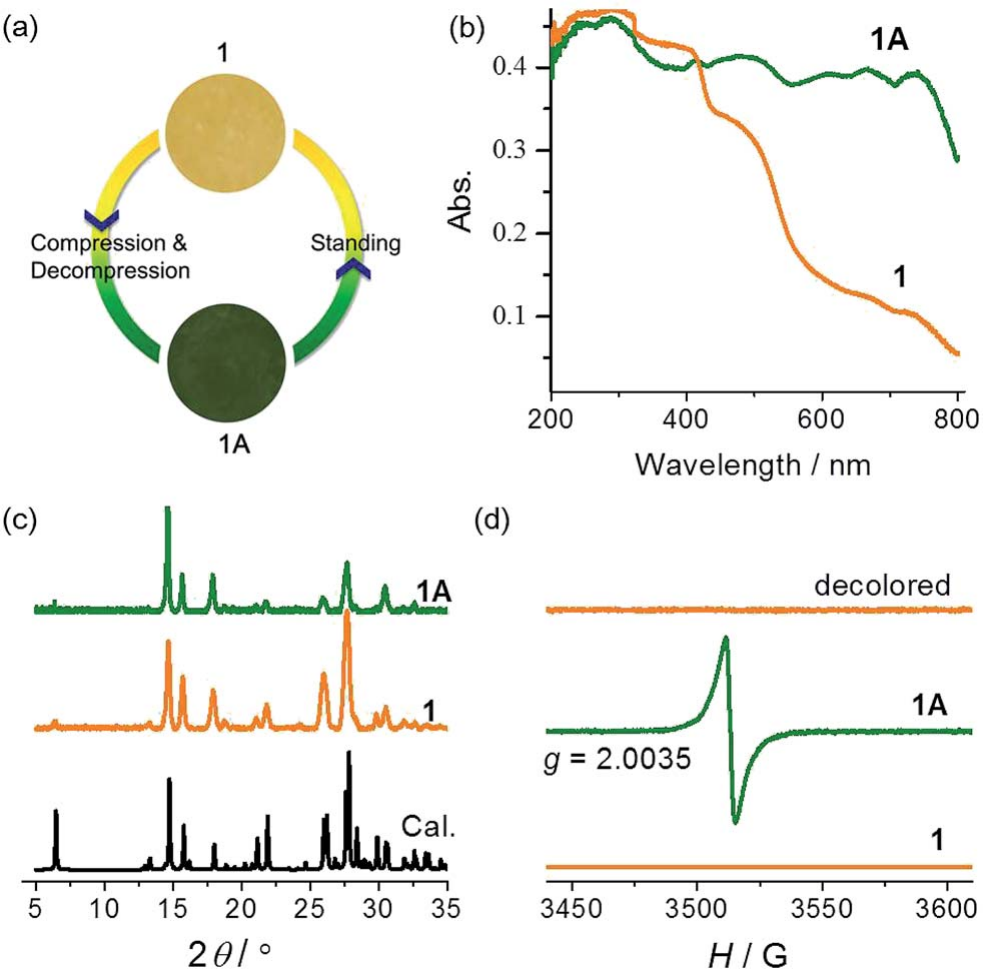
Photographs (a), UV-vis spectra (b), PXRD patterns ((c), *λ* = 1.5406 Å), and EPR spectra (d) of **1** and **1A**.

Compound **1** was synthesized as yellow crystals. No color change was observed when the crystals were ground. When preparing the KBr disc with a hydraulic press for IR spectroscopy, we unexpectedly obtained a green disc after release of pressure. The same color change was observed when pure samples of **1** were pressed under a pressure estimated at 2.8 GPa (Fig. 2a). A control experiment in the dark was performed to confirm that light has no contribution to the color change. The piezochromic phenomenon was confirmed by UV-vis diffuse reflectance spectra (Fig. 2b). The original sample of **1** displays intense absorption bands below 400 nm, with a shoulder band at about 470 nm. The visible-region absorption is responsible for the yellow color and attributable to the CT transition from the electron-rich carboxylate group to the electron-deficient viologen moiety.^22,30^ The green sample (**1A**) obtained after release of pressure shows much more intense absorption in the visible light region, with four maxima at 480, 610, 670 and 740 nm. The differences indicate that the compression–decompression process has caused dramatic changes in electronic structure. The powder X-ray diffraction (PXRD) profile of **1A** is very similar to that of **1** (Fig. 2c), with only some differences in relative intensity because of changes in preferential orientation. The observation suggests that there are no significant crystallographic differences between the original and final states. Furthermore, the IR spectra (Fig. S2†) of **1** and **1A** are essentially identical, so no significant difference is expected between the molecular structures of the two states. Considering the ability of the viologen moiety to form the V˙^+^ radical *via* one-electron transfer, we performed electron paramagnetic resonance (EPR) measurements to probe the piezochromic mechanism of **1** (Fig. 2d). It proved that **1** is EPR silent but **1A** presents a single strong signal with *g* = 2.0035, indicative of the presence of radicals. Therefore, piezochromism of **1** involves the formation of radicals through pressure-induced electron transfer. In other words, the compression–decompression process induces a redox chemical transformation from closed-shell to radical states. The two states under ambient pressure have no detectable differences in molecular and crystal structure but show distinctly different electronic structures.

**Fig. 3:**
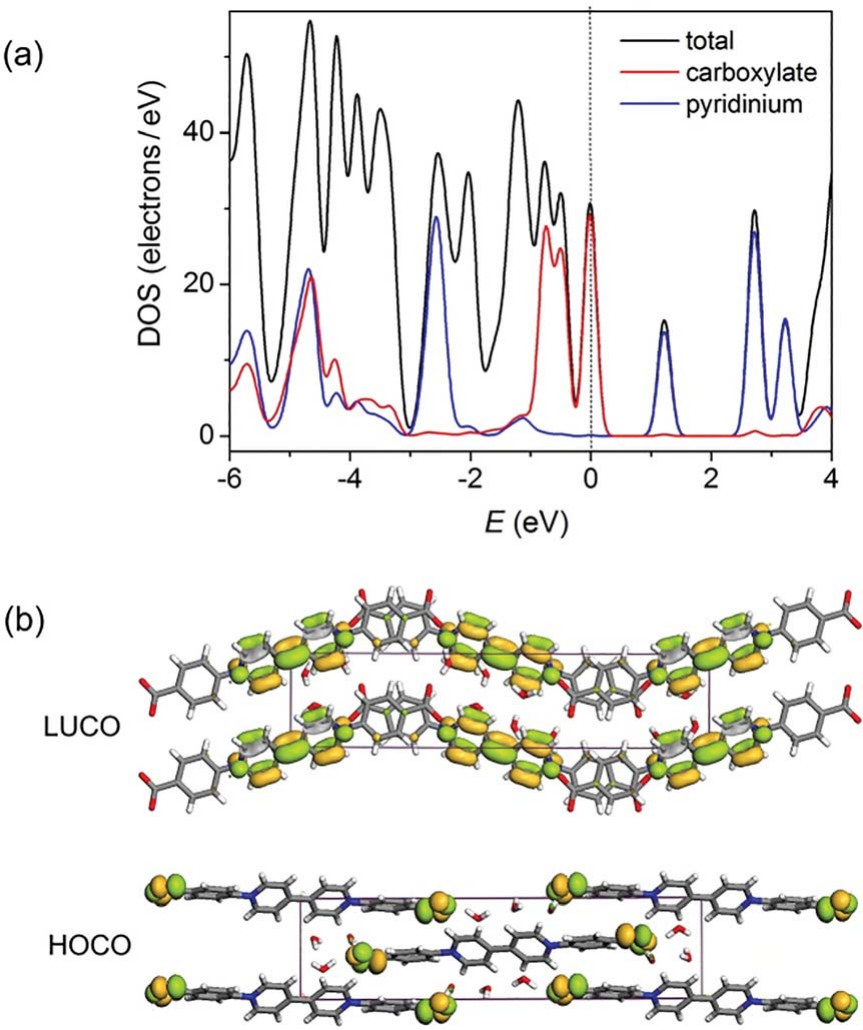
(a) Total and partial density of states calculated from single-crystal data of **1**. The Fermi level is located on 0 eV (dashed line). (b) Calculated ground-state frontier orbitals of **1**.

Density functional theory (DFT) calculations of the density of states (DOS) using the crystallographic data of **1** suggest that the top of the valence band and the bottom of the conduction band are overwhelmingly dominated by the carboxylate and pyridinium groups, respectively (Fig. 3a and S3†). Orbital analysis shows that the highest occupied crystal orbital (HOCO) arises almost solely from the nonbonding p orbitals of the carboxylate oxygen atoms, and the lowest unoccupied crystal orbital (LUCO) is dominated by the p–π* orbitals of the viologen moiety (Fig. 3b). Therefore, it is reasonable to assume that the electron transfer occurs from carboxylate oxygen to pyridinium, generating viologen and carboxylate radicals.

**Fig. 4:**
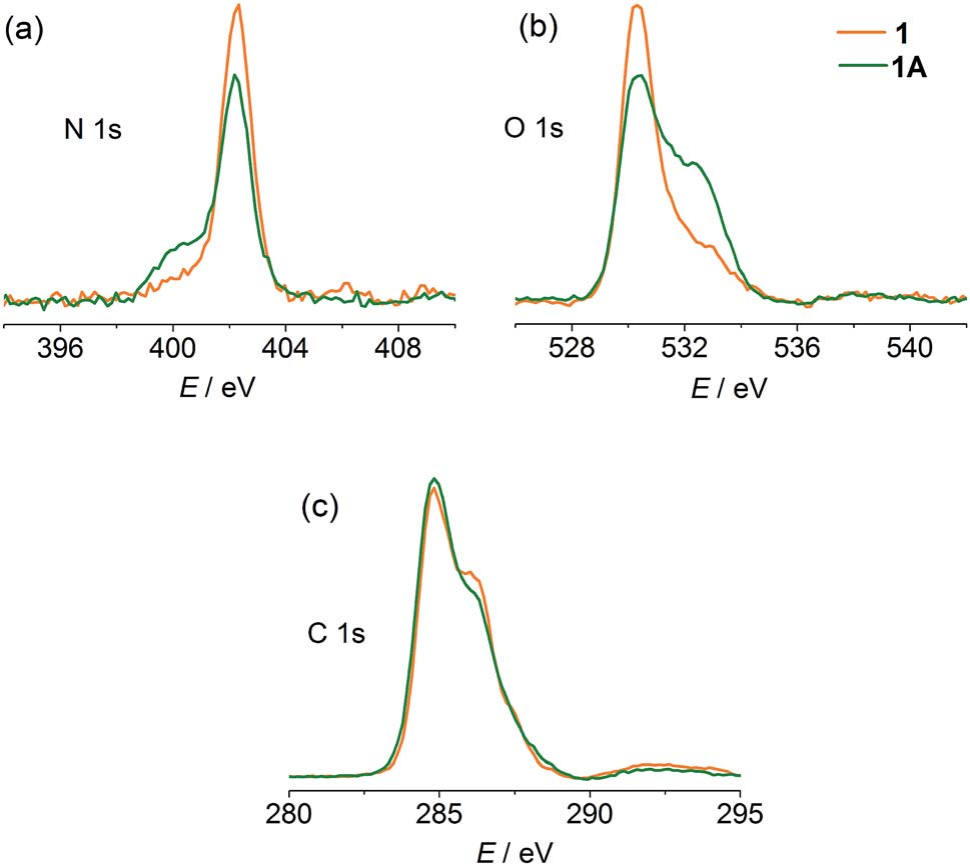
XPS spectra of **1** and **1A**.

X-ray photoelectron spectroscopy (XPS) was performed to gain experimental evidence for the direction of electron transfer. As shown in Fig. 4, the original state of the compound shows a single N 1s core-level peak at 402.3 eV due to the unique pyridinium. The carboxylate and water O atoms in the structure lead to a peak at 530.3 eV with a weak shoulder at 532.9 eV. After compression, a lower-energy shoulder (400.1 eV) appears in the N 1s spectrum, and the high-energy shoulder of the O 1s spectrum is significantly increased in intensity. The C 1s signals show a slight decrease in the high-energy shoulder. The phenomena are in good agreement with the occurrence of electron transfer from carboxylate to pyridinium.^7a,31^

The yellow and green colors can be reversibly switched. The pressure-induced green state is metastable. It completely recovers to yellow upon standing overnight, and the recovery can be accelerated by gentle heating below 90 °C (higher temperature causes dehydration of the compound; see the Hydrochromism section). The recovered sample is EPR silent (Fig. 2d), suggesting quenching of the radical. The reversible switching between the two states could be repeated for at least 10 cycles, with no apparent sign of fatigue (Fig. S4†). Notably, the recovery process is independent of the atmosphere and can occur in air, nitrogen and vacuum, so the possibility that radicals are quenched by molecular oxygen can be precluded. Radical quenching should proceed through spontaneous back electron transfer from viologen to carboxylate.

### Studies under hydrostatic pressure

The piezochromic properties of **1** were further investigated under hydrostatic pressure generated in diamond anvil cells (DACs). The DAC technology can not only provide isotropic gigapascal pressure but also allows us to study the color and spectral changes during compression and decompression.

**Fig. 5:**
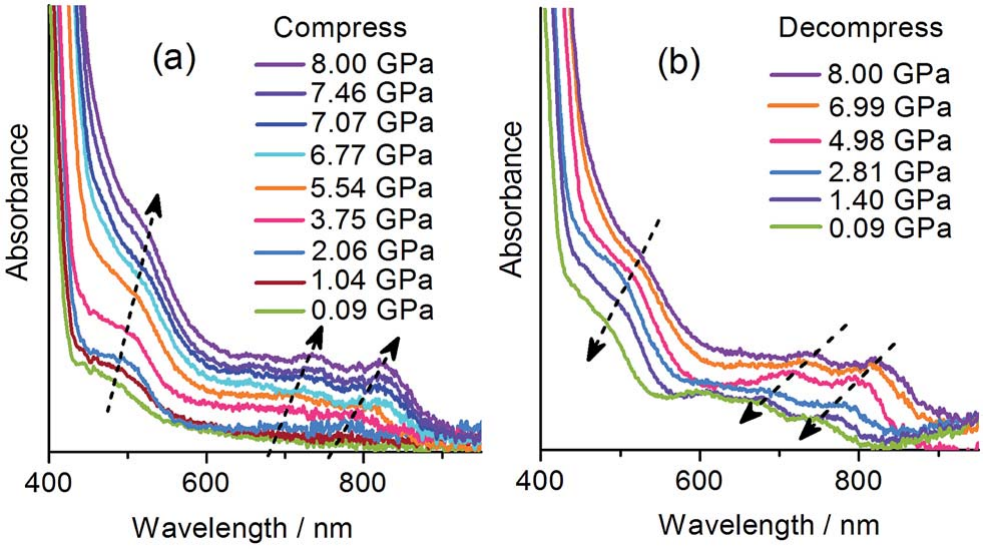
Pressure-dependent UV-vis spectra of **1**.

With the DAC equipment, we found that the pale yellow crystal turns red gradually as the pressure increases. No green color was observed in the compression process, but the crystal begins to show green tints when decompressed to about 2 GPa, and becomes green upon further decompression. UV-vis absorption spectra were recorded under various pressures using a single crystal in a DAC. As shown in Fig. 5a, under a relatively low pressure (0.09 GPa), the absorption in the visible region is dominated by the CT band at about 470 nm, as observed at atmospheric pressure. Upon compression above 2 GPa, new bands appear in the region of 600–800 nm, indicative of radical formation. As the pressure increases, the bands become more intense and show gradual bathochromic shifts. The increase in intensity could be due to the increasing concentration of radical species. The bathochromic shifts could be due to the modulating effects of pressure on intermolecular interactions and molecular conformation.^18*b*^ During decompression, the bands show a regular blue shift and decrease in intensity. However, even after complete removal of pressure, the radical signals still remain, with the maxima at essentially the same positions as observed for the green sample obtained after compression with a hydraulic press. The study indicates that the piezochromism of **1** under hydrostatic pressure also proceeds through an electron transfer mechanism. The color of the radical state is red under high pressure, consistent with the much stronger absorption below 600 nm, and it changes to green after decompression, in accordance with the shift in the multi-band visible-light absorption.

**Fig. 6:**
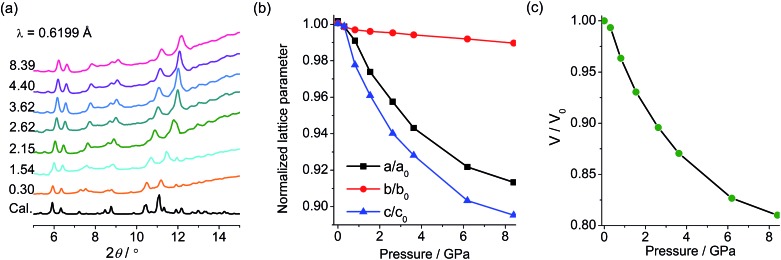
(a) Pressure-dependent XRD profiles of **1** with pressure given in GPa. (b) Variation of lattice parameters with pressure. (c) Variation of unit cell volume of **1** with pressure.

PXRD and IR studies have suggested that the final green state obtained after the removal of pressure shows no significant differences from the original yellow state in the molecular and crystal structures. To probe the possible structural change during the piezochromic process, *in situ* PXRD measurements in a DAC were performed using a synchrotron radiation source. As shown in Fig. 6a, under increasing pressure up to 8.4 GPa, the compound remains crystalline, and the diffraction peaks are systematically shifted to higher angles, which corroborates a volume contraction upon compression. The patterns have been indexed according to the crystallographic data of **1**. The refined unit cell parameters are plotted against pressure in Fig. 6b and c. As expected, *a*, *b*, *c* and *V* all contract under pressure. Under 8.4 GPa, the long axis (*b*) decreases by only 1.1% (0.31 Å), *a* and *c* decrease respectively by 9.0 and 10.7% (0.56 and 0.74 Å), and the cell volume decreases by 19% (227 Å^3^). The *in situ* PXRD study indicates that the piezochromic process does not involve crystallographic phase transitions.

In addition, *in situ* Raman scattering spectra were measured under 0–8.32 GPa. As shown in Fig. S5,† all peaks shift to higher frequencies, as is expected for molecules under high pressure. No new peak was detected and the spectrum was recovered concomitantly when pressure was removed. The results suggest that the compression/decompression process does not involve bond formation or cleavage in the pressure range investigated,^32^ as expected for the redox process of the viologen unit.

### Further insight into the piezochromic process

Photochromism in crystalline viologen-derived compounds usually involves intermolecular electron transfer, and the pathway for the transfer has been related to a short contact between the pyridinium N atom and the donor atom.^22,33^ With carboxylate donors, the N···O distance enabling photo-induced intermolecular electron transfer is usually around 3.5 Å.^7d,29^ No unequivocal criterion has been established, perhaps because the process could be influenced by the nature and various environmental factors of the donor and the acceptor. For piezochromism in viologen compounds, no previous example is available for comparison. The influencing factors for piezochromism could be different from those for photochromism, because pressure and light are disparate in nature and in the way they interact with solids. Nevertheless, shorter donor–acceptor distances should always be favorable for electron transfer, regardless of the stimulus. In this sense, we can envision that pressure could be a more powerful energy tool than light for induction of intermolecular electron transfer (and chromic phenomena) in viologen crystals. Light can be absorbed by individual molecules and have no direct effect on intermolecular distances, so it would not induce electron transfer if the donor and the acceptor were not placed at a favorable distance. Quite differently, pressure works directly on intermolecular distances, so it can proactively reduce the distance between the donor and the acceptor in favor of electron transfer. The fact that compound **1** is piezochromic but not photochromic (*vide infra*) could be an illustration of the power of pressure to induce intermolecular electron transfer.

**Fig. 7:**
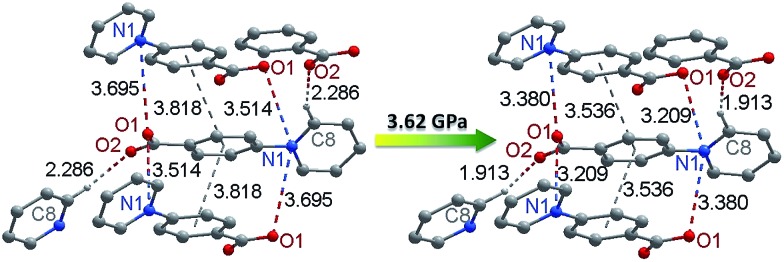
Pressure-induced changes of donor–acceptor contacts in **1** (the structure under high pressure was drawn according to the results of DFT geometry optimization).

To better understand the pressure-induced electron transfer, DFT geometry optimization using the cell parameters obtained from high-pressure XRD was performed to calculate the molecular packing structure under high pressure. The results show that the zwitterionic molecule is less twisted under pressure, with the angle between the pyridinium and phenyl rings being reduced from 33.8 to 21.9° under 3.62 GPa. The center-to-center distance between the π–π stacking benzene rings is reduced from 3.82 to 3.54 Å (Fig. 7). Accordingly, the intermolecular N···O distances between viologen and carboxylate moieties change from 3.51 and 3.70 Å at ambient pressure to 3.21 and 3.38 Å under 3.62 GPa. The significantly reduced donor–acceptor contacts could facilitate the electron transfer. Another possible pathway, which has been occasionally referred to in photochromic study, is the hydrogen bond (C8–H···O2) between the carboxylate oxygen and the pyridinium H atom adjacent to the N atom.^29*a*^ The H···O contact is significantly shortened from 2.29 to 1.91 Å under 3.62 GPa, while the C–H···O angle undergoes a very minor change (<1°).

It is difficult to definitively decide between inter- and intramolecular electron transfer. The above theoretical study supports intermolecular pathways more than intramolecular ones, because the molecular conformation remains twisted under pressure while the intermolecular donor–acceptor contacts are significantly reduced. This is consistent with the fact that the intermolecular pathway has been well established in photochromic studies of viologen compounds in both solution and solid phases. We performed further experiments to check whether piezochromism can occur in methyl viologen (MV^2+^) based systems, where only intermolecular pathways are possible. We obtained a solid by concentrating the mixture solution of [MV]Cl_2_ and benzoic acid in ethanol. The solid and a pure sample of [MV]Cl_2_ were compressed with a hydraulic press. No color change was observed for [MV]Cl_2_, but the solid obtained from [MV]Cl_2_ and benzoic acid turned from white to violet. The violet color and the intense absorption covering the region from 400 to 700 nm are typical of the MV˙^+^ radical.^22^ Although the structure is unclear, the radical can only be generated by intermolecular electron transfer (Fig. S6†). The simple experiments give us a clue to speculate that the piezochromism of **1** could proceed through intermolecular pathways. The study also demonstrates that pressure is a powerful tool to induce chromic phenomena *via* the intermolecular electron transfer mechanism. We can envision that piezochromism could be a common phenomenon for viologen-based molecular solids if appropriate donors are present. This is to be demonstrated by extended studies.

According to the above studies, the piezochromic process of **1** can be proposed as follows. Under high external pressure, the molecules in the lattice are forced to move closer. The intermolecular contacts between the electron-rich carboxylate group and the electron-deficient viologen moiety are shortened to favorable distances to induce electron transfer from carboxylate to viologen. This leads to a metastable state with two radical sites per molecule, one delocalized over the viologen moiety, the other over the carboxylate groups. There could be intermolecular radical–radical interactions, which, together with planar configuration of the viologen moiety, should help to stabilize the radical state. It could be assumed that the reverse electron transfer is slow and cannot keep pace with the recovery of the intra- and intermolecular parameters upon decompression. Therefore, although the molecular and crystal structure is completely recovered after removal of pressure, the radical state remains in a certain proportion. The back transfer process continues upon further standing at ambient pressure to finally regenerate the original state. Here, the key to the piezochromism is the formation of radicals *via* pressure-induced intermolecular electron transfer. The radical mechanism is different from those where pressure modulates π–π* and CT absorptions by strengthening intermolecular π overlapping, reducing intramolecular twisting;^8^ it is also different from the cases in which pressure induces bond cleavage/formation.^20,21^ Finally, it should be mentioned that not only electron transfer but also the gradual structural changes contribute to the piezochromic phenomena of **1**. Electron transfer leads to new absorption bands, and the gradual shifts of the bands during the compression/decompression process should be attributed to the modulation effects of pressure on intermolecular interactions and molecular conformation.

### Hydrochromism

**Fig. 8:**
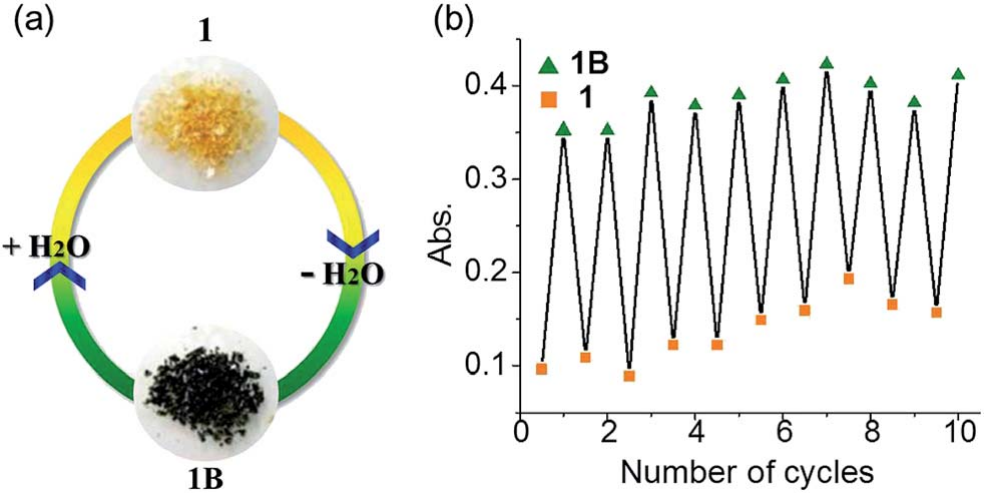
Hydrochromism of **1**. (a) Photographs. (b) Absorbance at *λ* = 728 nm in repeated dehydration–hydration cycles.

Many viologen derivatives are photochromic. In an initial test, a yellow sample of **1** turned green upon irradiation with a 300 W Xe lamp, which seemed to suggest a photochromic behavior. However, when the sample was cooled with jacket water to avoid heat accumulation from the Xe lamp, no color change was observed even under prolonged irradiation. This precludes photochromism but suggests a thermal effect. Indeed, heating **1** at 100 °C led to a green sample (Fig. 8).

**Fig. 9:**
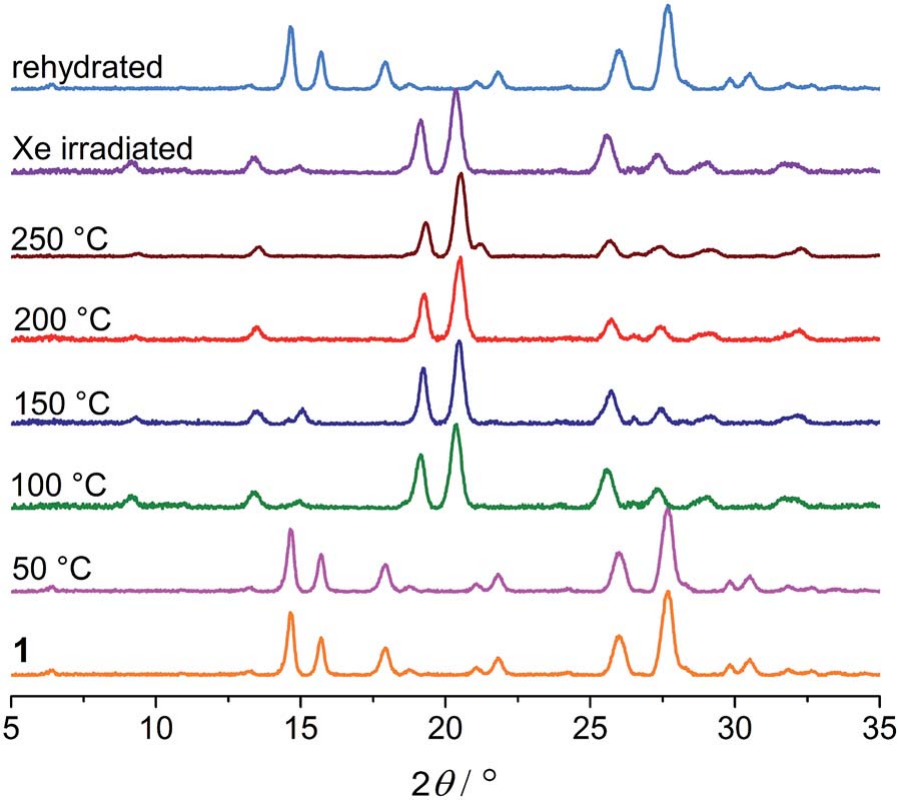
PXRD profiles of **1** heated at different temperatures, the sample subjected to Xe-light irradiation without cooling, and the rehydrated sample.

Thermogravimetric analysis (TGA), variable-temperature IR and PXRD measurements were performed to investigate other thermal effects on **1**. TGA and IR (Fig. S7 and S8†) suggest that **1** loses the water of crystallization upon heating up to 100 °C. As shown in Fig. 9, the PXRD profile of the anhydrous green phase is completely different from that of **1**. The new phase (hereafter denoted as **1B**) is crystalline and remains unchanged up to at least 250 °C. In addition, the XRD pattern of the green sample obtained by Xe-light irradiation without cooling is identical to that of **1B** (Fig. 9), confirming that the color change under the Xe lamp actually corresponds to the loss of water molecules due to the thermal effect of the lamp.

The UV-vis, EPR and XPS spectra of **1B** are very similar to those of **1A** (Fig. S9–S11†), so the dehydration-induced color change from **1** to **1B** is also due to the formation of radicals through one-electron transfer from carboxylate to viologen. Although the crystal structure of the anhydrous phase is unclear, it could be assumed that the loss of lattice water leads to closer crystal packing of the zwitterionic molecules so that the distance between electron donors and acceptors is reduced in favor of electron transfer.

The color change occurs readily by heating in the dark and hence electron transfer can occur without the aid of light stimulus. To clarify the role of heating, we have heated **1** at 110 °C in the atmosphere of water steam. No color change was observed, indicating that heating alone cannot induce electron transfer. Therefore, water release is the most essential factor, while heating just acts to facilitate water release. Particularly worth mentioning is that the color change also occurs if the compound is placed in a desiccator containing sodium hydroxide as desiccant for several hours (Fig. S12†). The phenomenon clearly confirms that dehydration can induce electron transfer even without the aid of heating, though heating can accelerate dehydration.

Viologen derived radicals can usually be quenched by molecular oxygen in air with concomitant color fading, and the quenching/fading process is usually thermally accelerated. Unexpectedly, the anhydrous radical state **1B** shows remarkable stability against temperature and oxygen. The green color persists upon heating up to 250 °C in dry air, even in a pure oxygen atmosphere. However, it can readily return to yellow in a moist atmosphere, and the color change occurs immediately if direct contact with liquid water is allowed. The yellow product obtained is EPR silent (Fig. S10†), and XRD studies indicate that the hydrate phase **1** is recovered (Fig. 9). It is worth noting that the reverse color change occurs without the aid of oxygen and heating, illustrating an innocent hydrochromic process. It can be assumed that insertion of water molecules back into the lattice sets the organic molecules apart from each other. The structural change induces back electron transfer so that radicals are quenched and thus color recovered.

The dehydration–hydration switched color change can be repeated for at least 10 cycles (Fig. 8b). Finally, it is worth mentioning that the anhydrous green phase **1B** does not change color when in contact with other solvents such as methanol, ethanol, acetonitrile and chloroform (either vapor or liquid), so the solvatochromic behavior is highly selective towards water.

## Conclusions

In summary, we have demonstrated interesting crystalline-state piezochromic and hydrochromic phenomena with a zwitterionic molecule containing anionic carboxylate as electron donor and cationic viologen as acceptor. The exertion of high pressure or the release of lattice water can induce electron transfer from carboxylate to viologen, generating radicals and thus leading to color change. The process is reversible *via* back electron transfer upon standing at ambient pressure or upon reabsorbing water. Despite their remarkably different nature, the physical stimuli (compression/decompression) and the chemical stimuli (dehydration/rehydration) have one thing in common: the ability to modulate intermolecular donor–acceptor contacts in favor of electron transfer. This work tells new chromic stories for the old family of viologen compounds well-known for electro-/photochromism, and presents the first example of organic mechanochromism and hydrochromism associated with radical formation through electron transfer. Most importantly, we demonstrated for the first time that the physical stimulus of pressure can induce single-electron transfer in viologen species, which used to be triggered by electricity and light. It is hoped that this fundamental finding can evoke further studies to explore the generality of pressure-induced electron-transfer in viologen derivatives and other redox-active organic species and to understand the physical and chemical factors influencing the processes. Considering the large family of viologen compounds and the diversity of redox-active organic compounds, the studies may open new ground and prospects in piezochromic organic materials and other related fields of piezochemistry. The results may also have implications for supramolecular studies dealing with stimulus-responsive organic systems.

## Experimental

### Materials and synthesis

4,4′-bipy, 2,4′-dinitrochlorobenzene, ethyl *p*-aminobenzoate and NaOH in AR grade were purchased commercially without further purification. Water was deionized and distilled before use. *N*,*N*′-Bis(2,4-dinitrophenyl)-4,4′-bipyridinium dichloride were prepared according to the literature.^34^

#### [H_2_bpybdc]Cl_2_

The synthetic routes of [H_2_bpybdc]Cl_2_ were shown in Scheme S1.† A mixture of *N*,*N*′-bis(2,4-dinitrophenyl)-4,4′-bipyridinium dichloride (1.1 g, 2.23 mmol) and ethyl 4-aminobenzoate (1.3 g, 7.87 mmol) was dissolved in 50% EtOH (30 ml) and refluxed for 24 h. After the mixture was cooled to room temperature, the solvent was evaporated and the residue dissolved in H_2_O. After filtering off the insoluble solid, the filtrate was washed with Et_2_O three times and evaporated to give a crude product of 1,1′-bis(4-ethoxycarbonylphenyl)-4,4′-bipyridinium dichloride ([Et_2_bpybdc]Cl_2_). The products were recrystallized from methanol/ethyl acetate (v/v, 1/2) to give a yellow powder. Yield: 0.35 g (30%).

[Et_2_bpybdc]Cl_2_ (0.35 g) dissolved in concentrated HCl (9 ml) was refluxed for 4 h. After cooling to room temperature, the precipitated solid product was filtered out, washed with water and dried under vacuum. [H_2_bpybdc]Cl_2_ was obtained as a pale yellow powder. Yield: 0.30 g (91%). IR (KBr, cm^–1^): 3506m, 3401m, 3112m, 3047w, 2572w, 1714s, 1633s, 1602s, 1546s, 1496s, 1444s, 1417s, 1380s, 1305m, 1174m, 1130w, 1106s, 865m, 836m, 798s, 769s, 723s, 696s, 640s, 572w, 545m, 520m, 501m. ^1^H NMR (400 MHz, D_2_O, ppm): 7.89 (d, *J* = 8 Hz, 4H), 8.25 (d, *J* = 8 Hz, 4H), 8.78 (d, *J* = 8 Hz, 4H), 9.42 (d, *J* = 8 Hz, 4H).

#### bpybdc·6H_2_O (**1**)

A solution of [H_2_bpybdc]Cl_2_ in hot water was neutralized using an aqueous NaOH solution (0.1 mol L^–1^). The solution was allowed to cool slowly to obtain yellow lamellate single crystals of **1** within a few hours. Yield: 92%. Elemental analysis calc. for C_24_H_28_N_2_O_10_ (*M* = 504.48): C, 57.14; H, 5.55; N, 5.55. Found: C, 56.82; H, 5.30; N, 5.34%. IR (KBr, cm^–1^): 3581w, 3122w, 2510w, 1699w, 1635s, 1606s, 1565s, 1542s, 1494s, 1438s, 1392s, 1375s, 1251w, 1230m, 1162w, 1137w, 1120w, 1037m, 1022m, 1004m, 875w, 840w, 777m, 690m, 534m, 470m. ^1^H NMR (400 MHz, D_2_O, ppm): 7.80 (d, *J* = 8 Hz, 4H), 8.08 (d, *J* = 8 Hz, 4H), 8.73 (s, 4H). Note: the 2,6 H atoms (neighboring to the N atom) of the pyridinium ring were not observed in the ^1^H NMR spectrum, owing to the fast H–D exchange with D_2_O under basic conditions. Consequently, the 3,5-H atoms appear as a singlet at 8.73 ppm.^35^ The zwitterionic compound is sparsely soluble in common solvents such as water, methanol, acetonitrile, and DMF.

### Crystal structure determination

Diffraction intensity data of **1** were collected at 293 K on a Bruker APEX II diffractometer equipped with graphite-monochromated Mo-Kα radiation (0.71073 Å) and a CCD area detector. Empirical absorption corrections were applied using the SADAB program.^36^ The structures were solved by the direct method and refined by the full-matrix least-squares method on *F* ^2^ using the SHELXL program,^37^ with anisotropic displacement parameters for all non-hydrogen atoms. The hydrogen atoms attached to carbon atoms were placed in calculated positions and refined using the riding model. The water hydrogen atoms were located from the difference Fourier map.

#### Crystal data for **1**

C_24_H_28_N_2_O_10_, *M*_r_ = 504.48, space group *P*2_1_/*c*, *a* = 6.2410(4), *b* = 27.3263(16), *c* = 6.9456(4), *β* = 99.4148(19)°, *T* = 296(2) K, *Z* = 2, *V* = 1168.57(12) Å^3^, *D*_c_ = 1.434 g cm^–3^, *μ* = 0.113 mm^–1^, *F*(000) = 532 and GOF = 1.092. 15 893 reflections collected, 2804 unique (*R*_int_ = 0.0289). *R*_1_ = 0.0442, w*R*_2_ = 0.1079 [*I* > 2*σ*(*I*)]. CCDC ; 1428498.†

### High-pressure experiments

For high pressure PXRD measurements, a sample was ground into fine powder and loaded into the sample chamber (100 μm in diameter) of a pre-indented stainless steel gasket in a diamond anvil cell (DAC) with 300 μm culets. A ruby sphere was then loaded to confirm pressure of the sample by using the ruby fluorescence.^38^ Silicone oil was used as a pressure-transmitting medium. *In situ* PXRD was performed at beamline 15U1 of Shanghai Synchrotron Radiation Facility (SSRF) using a wavelength of 0.6199 Å. 2D Debye–Scherrer diffraction rings from powder measurements were collected on a Mar345 image plate and integrated using the FIT2D software package.^39^ After each compression, the samples were allowed to equilibrate in the DAC until ruby fluorescence (pressure) was invariant. The pressure was verified again at the end of each diffraction experiment. The PXRD peaks were indexed according to the single-crystal data of **1**, and the unit cell parameters were refined using the least-squares method.

The *in situ* Raman scattering spectra were obtained in a DAC (500 μm culets) using a Renishaw 1000 spectrometer with a 532 nm excitation laser. The *in situ* UV-vis absorption measurements under high pressure were performed on an Ocean Optics QE65000 scientific-grade spectrometer with a DAC (300 μm culets) containing a single crystal of **1**. A ruby sphere was loaded to determine pressure by using the ruby fluorescence, and silicone oil was used as a pressure-transmitting medium.

All high pressure experiments were conducted at room temperature.

### Other physical measurements

^1^H NMR spectra were recorded on a Bruker Avance 400 MHz spectrometer. The FT-IR spectra were recorded in the range 500–4000 cm^–1^ using KBr pellets on a Nicolet NEXUS 670 spectrophotometer. Elemental analyses were performed on an Elementar Vario ELIII analyzer. Thermogravimetric analyses (TGA) were carried out on a Mettler Toledo TGA/SDTA851 instrument under flowing air at a heating rate of 10 °C min^–1^. Powder X-ray diffraction (PXRD) at ambient pressure was recorded on a Rigaku D/Max-2500 diffractometer at 35 kV, 25 mA for a Cu-target tube and a graphite monochromator. Optical diffuse reflectance (UV-vis) spectra were measured using a SHIMADZU UV-2700 spectrophotometer, with BaSO_4_ plates as references (100% reflection). Electron spin resonance (EPR) spectra were recorded on a Bruker Elexsys 580 spectrometer with a 100 kHz magnetic field in the X band at room temperature. X-ray photoelectron spectroscopy (XPS) studies were performed with a PHI 5000 Versaprobe spectrometer using Al Kα radiation (*λ* = 8.357 Å). To compensate for surface charging effects, all XPS spectra were referenced to the C 1s neutral carbon peak at 284.6 eV.

### Computation

Based on the X-ray crystallographic data of **1**, DFT (density functional theory) calculations were performed to analyze the density of states (DOS) and the frontier orbitals. The DMol^3^ module^40^ in the Materials Studio software package^41^ was used with fine accuracy for the numerical integration of the Hamiltonian and a fine (10^–6^ eV per atom) tolerance for SCF convergence. The DFT exchange–correlation potential was described by the Perdew–Burke–Eruzerhof (PBE) functional within the generalized gradient approximation (GGA).^42^ The Tkatchenko–Scheffler (TS) scheme was applied for dispersion corrections.^43^ All electrons were included in the calculations and the DNP (double numerical plus polarization) basis set was used with a fine orbital cutoff quality.

To investigate the effects of external pressure on molecular geometry and packing, the crystal structure of **1** under 3.62 GPa was optimized by DFT calculations using the CASTEP program.^44^ The cell parameters obtained from the high-pressure XRD experiment under 3.62 GPa were used without cell optimization. The optimization applied the GGA-PBE exchange–correlation functional and the TS dispersion-correction scheme. A fine optimization convergence level was selected, with an energy tolerance of 10^–5^ eV per atom, a maximum displacement of 0.001 Å, and a maximum force threshold of 0.03 eV Å^–1^. The norm-conserving pseudopotentials in reciprocal space were used for the electronic Hamiltonian, with an energy cut-off of 750 eV for the plane wave basis set and a SCF convergence tolerance of 10^–6^ eV per atom.

## References

[cit1] (a) Bamfield P. and Hutchings M. G., Chromic Phenomena: Technological Applications of Colour Chemistry, RSC, Cambridge, 2nd edn, 2010.

[cit2] Jaffe A., Lin Y., Mao W. L., Karunadasa H. I. (2015). J. Am. Chem. Soc..

[cit3] Byrne P. J., Richardson P. J., Chang J., Kusmartseva A. F., Allan D. R., Jones A. C., Kamenev K. V., Tasker P. A., Parsons S. (2012). Chem.–Eur. J..

[cit4] Sun J. K., Li W., Chen C., Ren C. X., Pan D. M., Zhang J. (2013). Angew. Chem., Int. Ed..

[cit5] Prashanthi S., Kumar P. H., Siva D., Lanke S. R., Rao V. J., Basak S., Bangal P. R. (2011). J. Phys. Chem. C.

[cit6] Harada J., Fujiwara T., Ogawa K. (2007). J. Am. Chem. Soc..

[cit7] Zhang Z. J., Xiang S. C., Guo G. C., Xu G., Wang M. S., Zou J. P., Guo S. P., Huang J. S. (2008). Angew. Chem., Int. Ed..

[cit8] Johnstone R. D. L., Allan D., Lennie A., Pidcock E., Valiente R., Rodriguez F., Gonzalez J., Warren J., Parsons S. (2011). Acta Crystallogr., Sect. B: Struct. Sci..

[cit9] Another subclass of mechanochromism is tribochromism, for which the stimulus is grinding or attrition (ref. 1a). Grinding and attrition are obviously different from pressure in the usual sense and can cause various effects besides compression. In the literature, however, the color change in response to grinding has been confusingly called “piezochromism” in many cases. Actually, tribochromism and real piezochromism do not necessarily coexist or share the same color and mechanism, and the different stimuli have very different implications for applications. Therefore, there is a need to clearly discriminate between them. The need is growing as the number of such materials is increasing rapidly. In this article, we do not include tribochromism when referring to piezochromism. Nagura K. Saito S. Yusa H. Yamawaki H. Fujihisa H. Sato H. Shimoikeda Y. Yamaguchi S., J. Am. Chem. Soc., 2013, 135 , 10322 –10325 .23815169

[cit10] Lang J. M., Dreger Z. A., Drickamer H. G. (1993). J. Phys. Chem..

[cit11] Gutierrez V. B., Cornu L., Demourgues A., Gaudon M. (2015). ACS Appl. Mater. Interfaces.

[cit12] Bray K. L., Drickamer H. G., Schmitt E. A., Hendrickson D. N. (1989). J. Am. Chem. Soc..

[cit13] Zahner J. C., Drickamer H. G. (1960). J. Chem. Phys..

[cit14] Pinkowicz D., Rams M., Misek M., Kamenev K. V., Tomkowiak H., Katrusiak A., Sieklucka B. (2015). J. Am. Chem. Soc..

[cit15] Chung W., Shibaguchi H., Terao K., Fujiki M., Naito M. (2011). Macromolecules.

[cit16] Sato T., Yagi T., Tajima H., Fukuda T., Yamamoto T. (2008). React. Funct. Polym..

[cit17] Dreger Z. A., Drickamer H. G. (1997). J. Phys. Chem. A.

[cit18] Meng X., Qi G., Zhang C., Wang K., Zou B., Ma Y. (2015). Chem. Commun..

[cit19] Wang L., Wang K., Zou B., Ye K., Zhang H., Wang Y. (2015). Adv. Mater..

[cit20] Liu L., Wang A., Wang G., Munyentwari A., Zhou Y. (2015). J. Lumin..

[cit21] Lekin K., Phan H., Winter S. M., Wong J. W. L., Leitch A. A., Laniel D., Yong W., Secco R. A., Tse J. S., Desgreniers S., Dube P. A., Shatruk M., Oakley R. T. (2014). J. Am. Chem. Soc..

[cit22] Monk P. M. S., The Viologens: Physicochemical properties, synthesis and applications of the salts of 4,4'-bipyridine, Wiley-VCH Verlag GmbH, 1999.

[cit23] McGonigal P. R., Deria P., Hod I., Moghadam P. Z., Avestro A.-J., Horwitz N. E., Gibbs-Hall I. C., Blackburn A. K., Chen D., Botros Y. Y., Wasielewski M. R., Snurr R. Q., Hupp J. T., Farha O. K., Stoddart J. F. (2015). Proc. Natl. Acad. Sci. U. S. A..

[cit24] Balzani V., Credi A., Raymo F. M., Stoddart J. F. (2000). Angew. Chem., Int. Ed..

[cit25] Sun J. K., Jin X. H., Cai L. X., Zhang J. (2011). J. Mater. Chem..

[cit26] Burneo I., Stylianou K. C., Rodriguez-Hermida S., Juanhuix J., Fontrodona X., Imaz I., Maspoch D. (2015). Cryst. Growth Des..

[cit27] Park D.-H., Park B. J., Kim J.-M. (2016). Acc. Chem. Res..

[cit28] Sheng L., Li M., Zhu S., Li H., Xi G., Li Y. G., Wang Y., Li Q., Liang S., Zhong K., Zhang S. X. A. (2014). Nat. Commun..

[cit29] Toma O., Mercier N., Allain M., Kassiba A. A., Bellat J.-P., Weber G., Bezverkhyy I. (2015). Inorg. Chem..

[cit30] Yoshikawa H., Nishikiori S.-I. (2005). Dalton Trans..

[cit31] Sampanthar J. T., Neoh K. G., Ng S. W., Kang E. T., Tan K. L. (2000). Adv. Mater..

[cit32] Tao Y., Dreger Z. A., Gupta Y. M. (2014). Vib. Spectrosc..

[cit33] Leblanc N., Bi W., Mercier N., Auban-Senzier P., Pasquier C. (2010). Inorg. Chem..

[cit34] Kamogawa H., Sato S. (1991). Bull. Chem. Soc. Jpn..

[cit35] Rieger A. L., Edwards J. (1985). J. Org. Chem..

[cit36] Sheldrick G. M., SADABS, Program for Empirical Absorption Correction, University of Göttingen, Göttingen, Germany, 1996.

[cit37] Sheldrick G. M., SHELXTL, Bruker Analytical X-ray Instruments Inc., Madison, Wisconsin, USA, 1998.

[cit38] Mao H. K., Xu J., Bell P. M. (1986). J. Geophys. Res., B.

[cit39] Hammersley A. P., Svensson S. O., Hanfland M., Fitch A. N., Hausermann D. (1996). High Pressure Res..

[cit40] Delley B. (2000). J. Chem. Phys..

[cit41] Materials Studio. version 5.0.0. Accelrys Software Inc., San Diego, CA, 2009.

[cit42] Perdew J. P., Burke K., Ernzerhof M. (1996). Phys. Rev. Lett..

[cit43] Tkatchenko A., Scheffler M. (2009). Phys. Rev. Lett..

[cit44] Clark S. J., Segall M. D., Pickard C. J., Hasnip P. J., Probert M. J., Refson K., Payne M. C. (2005). Z. Kristallogr..

