# Supplementary material for: Piezochromism and hydrochromism through electron transfer: new stories for viologen materials
Source: Chem Sci. 2016 Dec 22;8(4):2758–68. doi: 10.1039/c6sc04579k (PMC5426459; doi:10.1039/c6sc04579k)
Supplement: Supplementary file 1 [file SC-008-C6SC04579K-s001.pdf]

Electronic Supplementary Material (ESI) for Chemical Science.
This journal is © The Royal Society of Chemistry 2016

Electronic Supplementary Information (ESI)

Piezochromism and Hydrochromism through Electron Transfer: New Stories for Viologen Materials

Qi Sui, Xiang-Ting Ren, Yu-Xiang Dai, Kai Wang, Wen-Tao Li, Teng Gong, Jia-Jia Fang, Bo Zou, En-Qing Gao, and Lin Wang**

Supplementary Scheme.. S2

Supplementary Tables.. S3

Supplementary Figures.. S4

Supplementary Scheme

Scheme S1. Synthesis of $[H_2bpybdc]Cl_2$

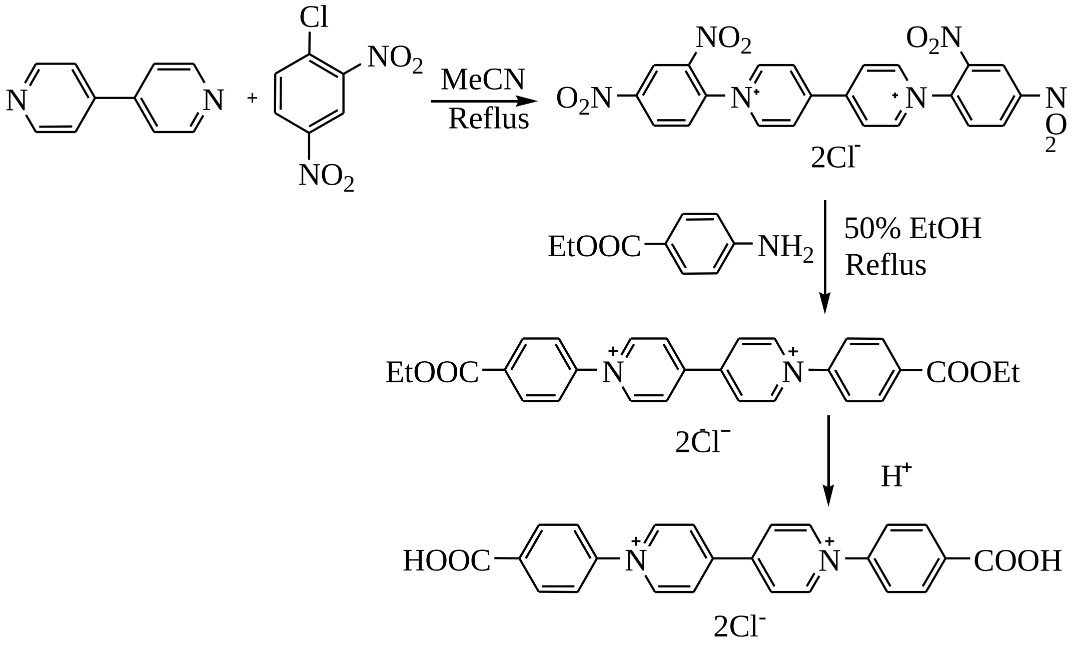

Supplementary Tables

Table S1. Hydrogen bonds of **1**.

	D-H	H···A	D···A	< (DHA)
C8-H8A-O2	0.93	2.29	3.168(2)	158.1
C9-H9A-O4	0.93	2.21	3.081(9)	154.9
C9-H9A-O4'	0.93	2.30	3.214(9)	167.7
C11-H11A-O4	0.93	2.50	3.390(9)	160.5
C11-H11A-O4'	0.93	2.52	3.445(8)	174.8
O4-H4B-O1	0.89(2)	1.86(1)	2.715(8)	158(3)
O4'-H4B-O1	0.90 (2)	1.86(1)	2.732(7)	161(2)
O4-H4C-O5'	0.87(2)	1.94(1)	2.792(12)	166(3)
O4'-H4C-O5	0.87(2)	1.99(2)	2.726(8)	142(2)
O3-H3B-O1	0.90(2)	1.86(2)	2.760(2)	178(2)
O3-H3C-O2	0.89(3)	1.86(3)	2.753 (2)	174(2)
O5-H5A-O4'	0.88 (2)	1.89(3)	2.759(14)	169(5)
O5'-H5'B-O3	0.86(2)	2.19(2)	3.013(7)	159(5)
O5-H5B-O3	0.88(2)	1.74(2)	2.608(5)	169(5)

Table S2 Lattice parameters of **1** under different external pressure.

Pressure / GPa	*a*	*b*	*c*	*β*	V / Å^3
0[a]	6.241	27.326	6.946	99.415	1168.6
0	6.243	27.326	6.935	99.435	1167.2
0.3	6.224	27.269	6.926	99.486	1159.5
0.81	6.176	27.225	6.779	99.353	1124.6
1.54	6.069	27.199	6.662	99.072	1086.1
2.62	5.967	27.178	6.518	98.499	1045.6
3.62	5.878	27.148	6.435	98.320	1016.1
6.19	5.745	27.087	6.263	98.104	964.8
8.4	5.693	27.026	6.208	98.034	945.7

[a] From single-crystal X-ray analysis. Others are from powder diffraction under different pressure.

Supplementary Figures

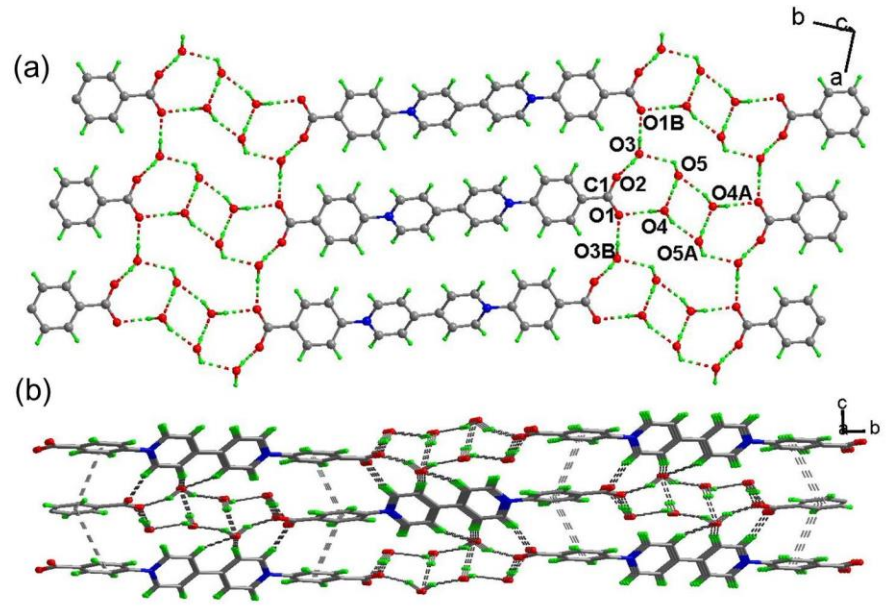

Fig. S1 (a): 2D hydrogen-bonded layer. (b): The 3D structure of **1** via π-π stacking and hydrogen-bonds. Symmetry code: A = 1-x, -y, 1-z; B = 1+x, y, z.

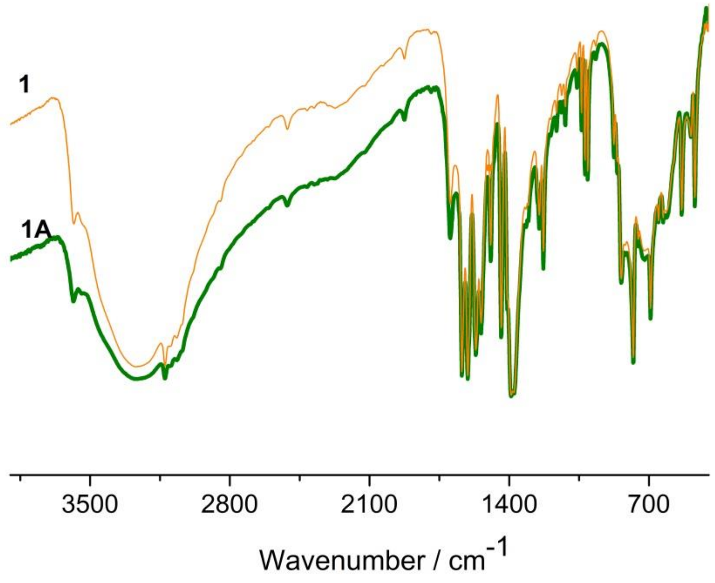

Fig. S2 IR spectra of **1** and **1A**.

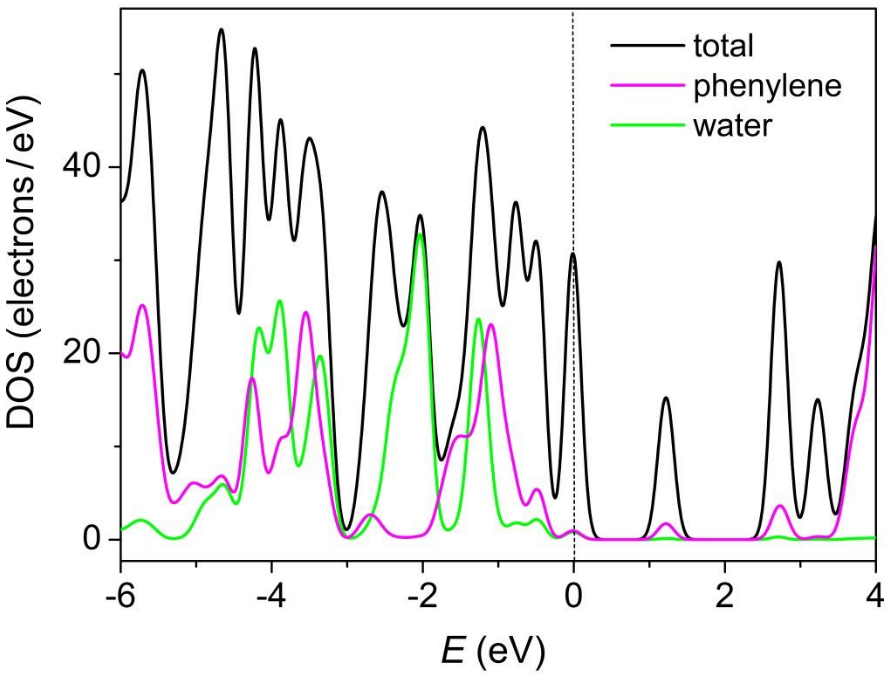

Fig. S3 Total and partial density of states calculated from single-crystal data of **1**.

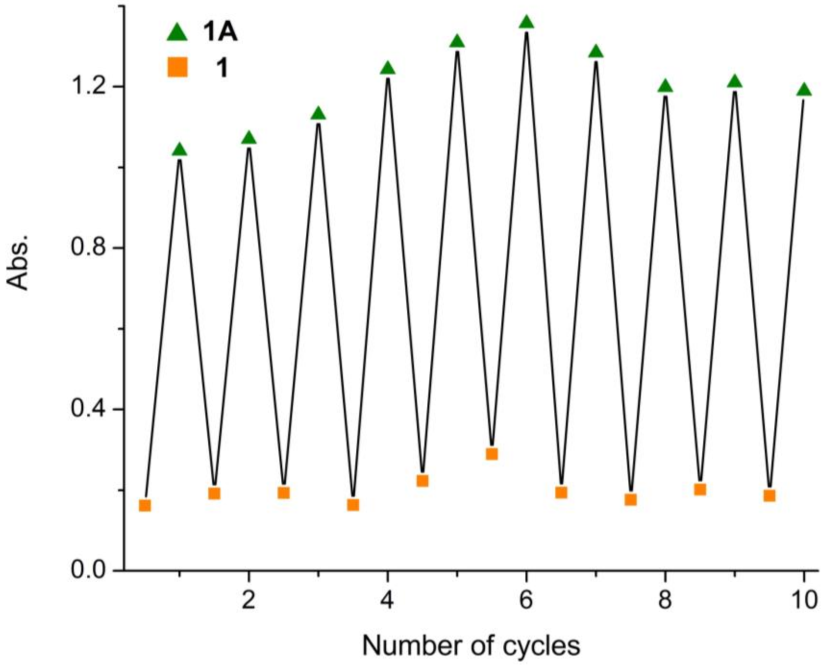

Fig. S4 The repeated cycles of **1** and **1A** (based on absorbance at λ = 739 nm).

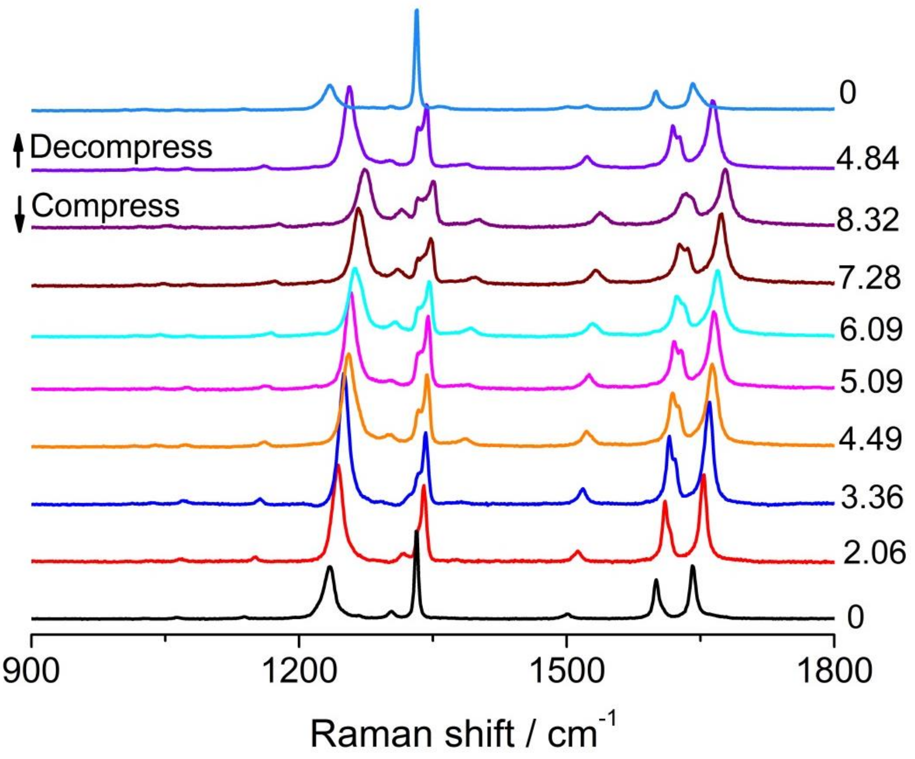

Fig. S5 The *in-situ* Raman spectra of **1** with pressure given in GPa.

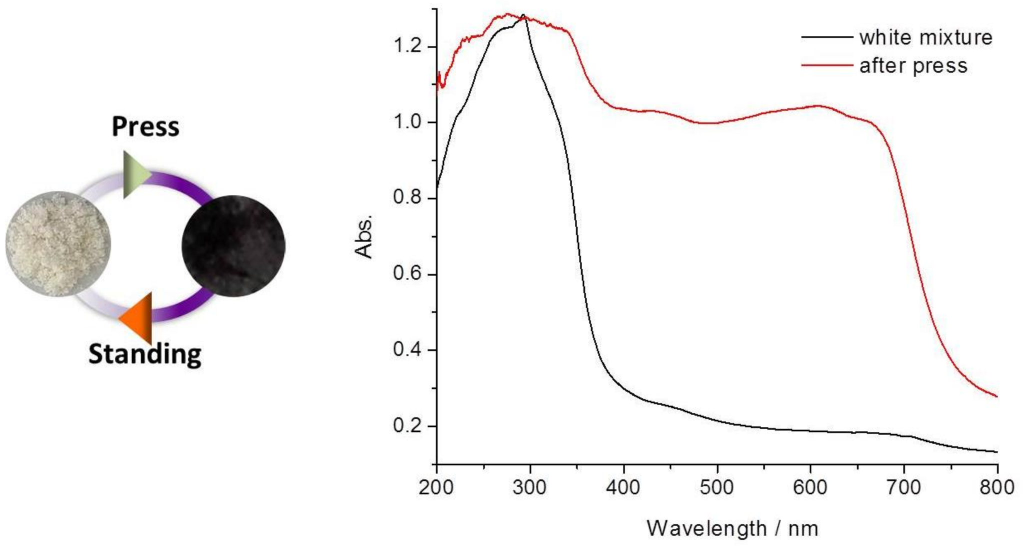

Fig. S6 The photographs and UV-vis spectra of a mixture of $MVCl_2$ and benzoic acid.

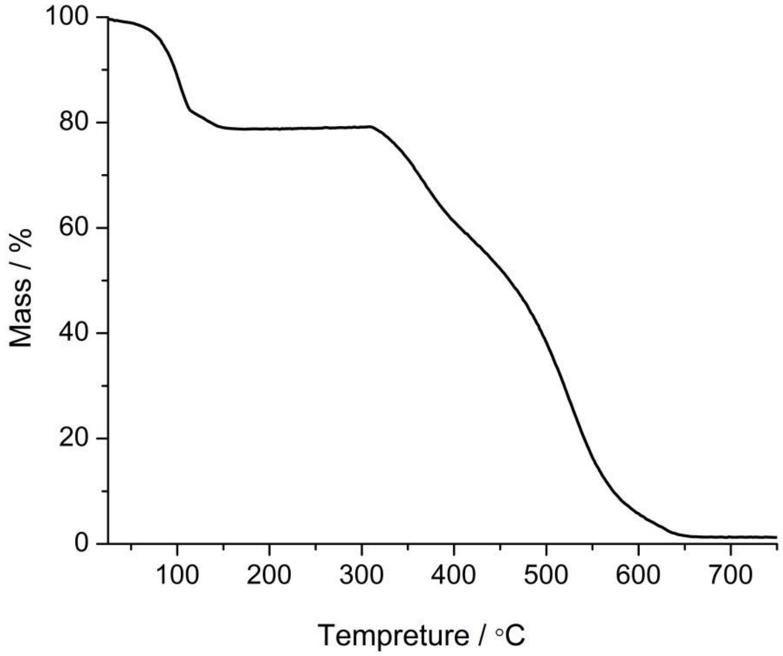

Fig. S7 Thermogravimetric plot of **1**.

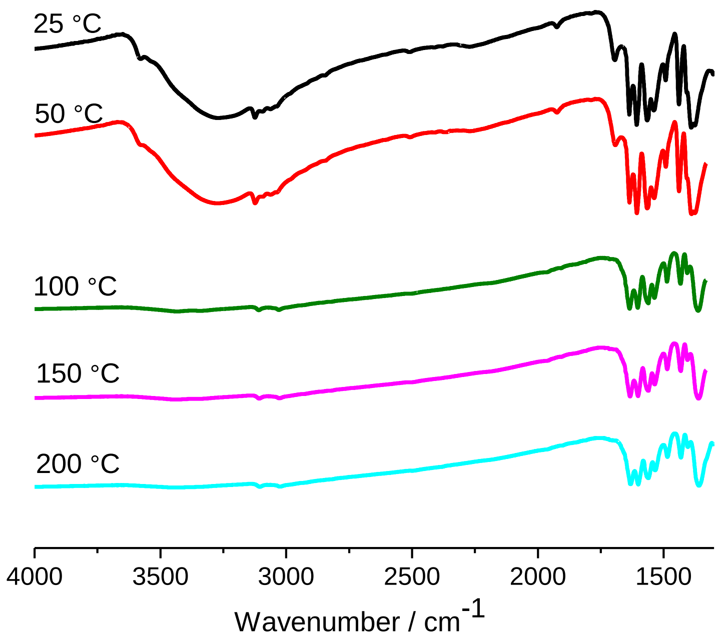

Fig. S8 *In-situ* IR of **1** at different temperature.

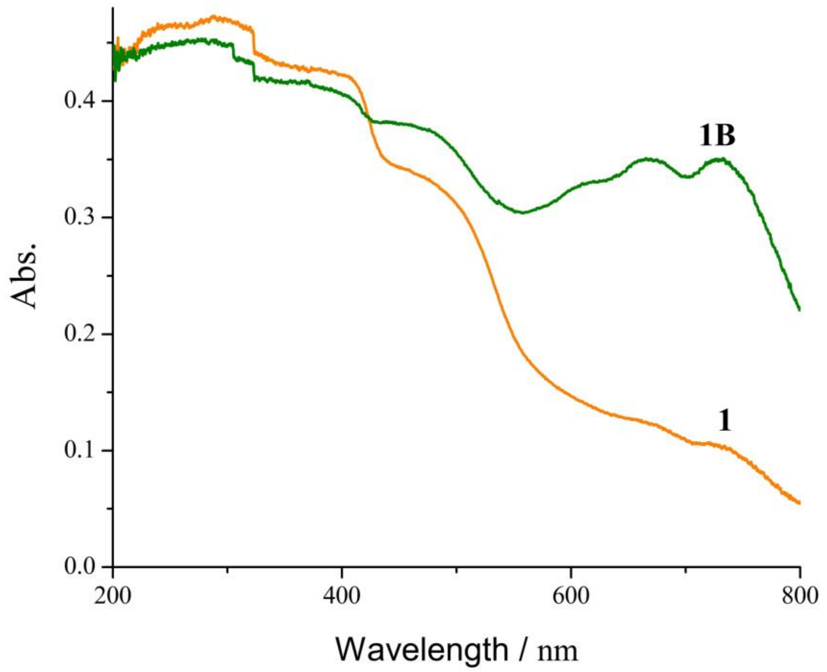

Fig. S9 UV-vis spectra of **1** and **1B**.

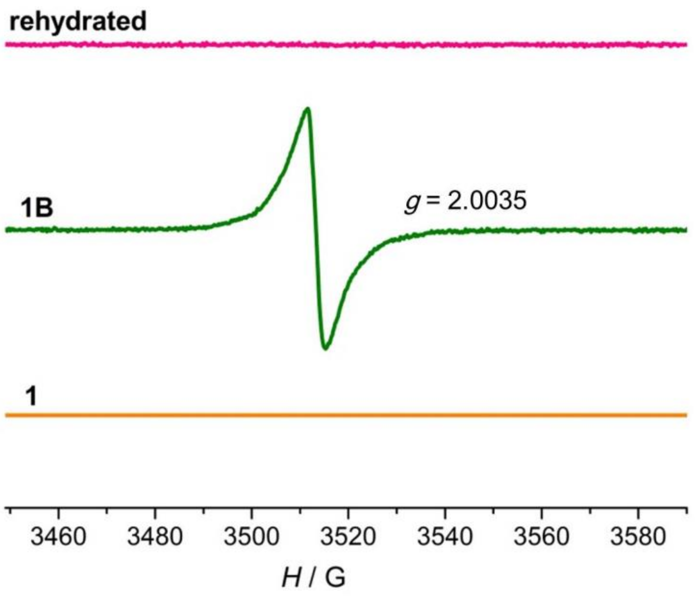

Fig. S10 EPR spectra of **1** and **1B**.

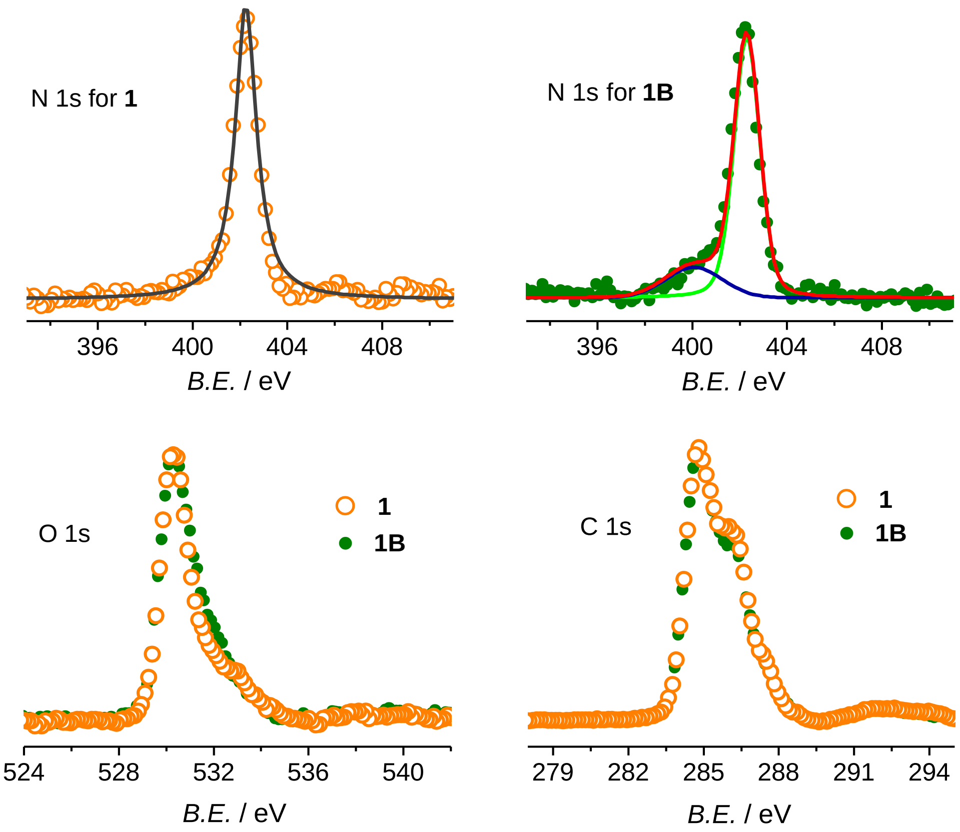

Fig. S11 XPS spectra of **1** and **1B**. The solid lines in the N 1s spectra depict the fitted curves.

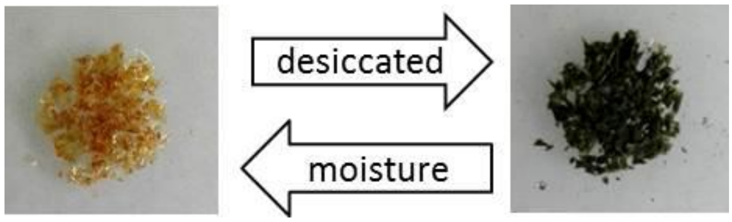

Fig. S12 The photographs of **1** before and after placed in a desiccator.
